# A cross-sectional survey of fertility knowledge in obstetrics and gynecology residents

**DOI:** 10.1186/s40738-020-00091-2

**Published:** 2020-12-09

**Authors:** Leah May Roberts, Rashmi Kudesia, Huaqing Zhao, Shaliz Dolan, Marisa Rose

**Affiliations:** 1 Department of Obstetrics, Gynecology and Reproductive Sciences, Temple University Hospital at the Lewis Katz School of Medicine, 3401 N Broad Street, Philadelphia, PA 19102 USA; 2 Division of Reproductive Endocrinology & Infertility, CCRM Houston, Houston, TX USA; 3 Division of Reproductive Endocrinology & Infertility, Houston Methodist Hospital, Houston, TX USA; 4 Department of Clinical Sciences, Temple University Hospital at the Lewis Katz School of Medicine, Philadelphia, PA USA

**Keywords:** Fertility, Fertility awareness, Infertility, Medical education, Residency education

## Abstract

**Background:**

To evaluate fertility knowledge among current Obstetrics and Gynecology (OB-GYN) residents using a recently published validated instrument, the Fertility and Infertility Treatment Knowledge Score (FIT-KS).

**Methods:**

OB-GYN residents in the United States were recruited through an email to all residency coordinators nationwide. They were asked to voluntarily respond to a short questionnaire including demographic information and the FIT-KS instrument, through an online survey platform. Of approximately 5000 OB-GYN residents in the country, 177 responded.

**Results:**

The sample was 91% female, with 69% between the ages of 26 and 30. Participants evenly represented all 4 years of training. Mean FIT-KS score was 21.2 (73% correct; range 17–26). No statistically significant differences were noted across the level of training. Several knowledge gaps were noted. Residents could define the common assisted reproductive technologies; however overestimated their success rates per cycle.

**Conclusions:**

Substantial gaps exist in fertility knowledge among OB-GYN residents, with understanding of male fertility and success rates of Assisted Reproductive Technologies (ART) being particularly limited. Knowledge of fertility does not change throughout residency training, demonstrating consistent gaps in fertility knowledge. Knowledge during post graduate year (PGY)-1 year is consistent with mean scores found in prior research in Internal Medicine residents (65%), as well as a cohort of female medical students and obstetrics and gynecology residents and fellows (64.9%) (Fertil Steril 108:711-7, 2017; Fertil Steril 110:e239, 2018).

**Supplementary Information:**

The online version contains supplementary material available at 10.1186/s40738-020-00091-2.

## Background

Age related fertility decline has been highlighted in recent years in the popular media, however misconceptions still exist among the general public. Approximately 48.5 million couples worldwide experience infertility but the majority of the population does not understand natural fertility and age related fertility decline [1]. Prior research has demonstrated consistently low rates of fertility knowledge in international populations, in reproductive-aged women, and across educational spectra [2–4]. Until recently, however, there has not been an instrument validated in the U.S. for measuring fertility knowledge.

In the general population, criteria for infertility diagnosis are met by approximately 12.5% of women [5]. Women with higher educational attainment and occupational status were more likely to have experienced infertility [5]. We know that there is a delay in childbearing among female physicians, especially for surgeons [6]. On average, female physicians were found to have their first child 7.4 years later than the general population [6]. Thoracic surgeons wait even longer, at 9 years later [7]. A later start to family-building has been shown to decrease family size [8, 9]. Postponing parenthood and attempts at pregnancy is also associated with a higher rate of adverse pregnancy outcomes [9]. Women with higher education are also known to be more likely to underachieve their fertility intentions than those who do not pursue higher education [7, 10–12].

In one recent study, 24% of female physicians who tried to conceive were formally diagnosed with infertility, of whom 21.7% were ultimately unable to conceive [6]. A substantial portion (43.1%) of those diagnosed with infertility were “quite a bit” to “very much” surprised at their diagnosis [6]. Female urologists with successful births utilized assisted reproductive technologies (ART) at almost ten times (OR 9.77; 95% CI 5.91 to 16.16) the rate of the general population [13]. Thoracic surgeons have also been shown to utilize ART at a higher rate than the general population [7].

If, as these data suggest, female physicians have knowledge gaps about their own fertility, it also stands to reason that they may be unable to adequately counsel their patients on this topic. As such, this study aims to evaluate whether obstetrics and gynecology residents are appropriately knowledgeable about natural fertility and age related fertility decline.

## Materials and methods

Permission was received to use the Fertility and Infertility Treatment Knowledge Score (FIT-KS). The FIT-KS is a web-based survey, with 29 multiple-choice items. It assesses knowledge of natural fertility (21 questions) as well as infertility treatment (8 questions). It has previously been validated in both reproductive-aged women in the US as well as in female medical trainees. The correct answers were based on the latest Society for Assisted Reproductive Technology (SART) data from the year of creation of the survey (2014).

Institutional review board deemed the study exempt through Temple University College of Medicine (#25003). All participants gave written informed consent through the survey, and risk was deemed to be minimal.

In April of 2018, the research team emailed all Obstetrics and Gynecology residency directors with the request to forward the web-based survey to their residents through email. Two follow-up reminder emails were sent over April and May 2018. The survey was hosted by Survey Monkey, and at the end of the survey respondents were able to click on a separate url not linked to their original survey to be entered into a lottery for 1 of 4 $25 Amazon gift certificates. Our response rate calculation is imprecise as the exact number of residents who actually received the recruitment email from their residency directors is unknown, however in number of responses it was consistent with other surveys performed of this population. Of approximately 5000 active OB-GYN residents in the country, 177 responded.

Statistical analysis was performed using either an Analysis of Variance (ANOVA) or two sample t tests as appropriate for the data set. Bartlett’s test was used to verify that variances were equal across the samples, in cases where ANOVA was used, such as whether knowledge differed by year of residency training. All statistical analysis was performed in Stata 14.0 (StataCorp LP., College Station, TX). Of the residents who opened the survey, 11 failed to complete all questions and were discarded from the analysis.

The FIT-KS score was calculated by dividing the number of questions answered correctly but the total number of questions – 29. The higher the score, the more answers were correct.

In designing this project, an attempt was made to avoid bias by several methods. To avoid bias in our question design, we used a previously validated survey instrument, the FIT-KS. For further information on this validation process, please refer to Dr. Kudesia’s previously published paper in Fertility and Sterility [14].

## Results

### Demographics and fertility knowledge (Table 1)

Of approximately 5000 OB-GYN residents in 255 programs in the United States, there were 177 residents who responded to the survey. Of these, 166 completed all questions in the survey. The other 11 residents opened the study, but did not complete all questions. The demographics of those who opened but did not complete all questions were consistent with the other responses from the survey per analysis by our statistician, and they were excluded from the final analysis. We do not know what proportion of respondents came from which residency programs.

**Table 1:** Demographics and FIT-KS Score

Demographics	Number	Mean FIT-KS Score
PGY1	40	20.73 (71%; range 17–25)
PGY2	47	21.64 (75%; range 19–25)
PGY3	39	20.95 (72%; range 18–24)
PGY4	40	21.23 (73%; range 17–26)
**Gender**		
Male	13	20.57 (72%; range 18–24)
Female	153	21.20 (73%; range 17–26)
**Age**		
26–30	114	21.08 (73%; range 17–25)
31–35	50	21.32 (74%; range 18–25)
36–40	2	21.50 (74%; range 21–22)

Ninety-one percent of respondents were female. Sixty-nine percent were between the ages of 26–30. They represented an equal distribution between all four levels of training, with 40, 47, 39 and 40 representing each year of study (*n* = 166).

The difference in mean score of each year of study was not statistically significant. In total, the highest score available was 29. Post-graduate year (PGY)-1 residents received a mean score of 20.73 (71%; range 17–25), PGY-2 a mean score of 21.64 (75%; range 19–25), PGY-3 a mean score of 20.95 (72%; range 18–24), and PGY-4 a mean score of 21.23 (73%; range 17–26). Gender was also not a significant predictor of score: females’ average score was 21.20 (73%; range 17–26) and males’ average scores was 20.57 (71%; range 18–24). Age category also failed to predict score, with 26–30 scoring 21.08 (73%; range 17–25), 31–35 scoring 21.32 (74%; range 18–25), and 36–40 scoring 21.50 (74%; range 21–22) (*p* = 0.23).

Thirty-three percent stated they were not concerned about their own future fertility, and level of concern did not predict average FIT-KS score; those unconcerned about future fertility had a mean score of 21.36 (74; range 17–26%), while those with concerns scored an average of 21.15 (73%; range 17–26) (*p* = 0.57).

The majority of residents (158, 95%) stated that they had ever discussed fertility with their patients. Mean score of those who did discuss fertility was 21.21 (73%; range 17–26), compared to 21.60 (75%; range 20–26) for those who did not, *p* = 0.69. One hundred and thirty-three (82%) stated that they felt comfortable discussing fertility with their patients, with a mean score of 21.29(73%; range 17–26), compared to 20.9 (72%; range 17–24) for those who did not feel comfortable discussing fertility with their patients, *p* = 0.41. Seventeen respondents currently have children, with a mean score of 21.59 (74%; range 18–25), not significantly higher than those who did not at 21.18 (73%; range 17–26), *p* = 0.45.

### Gaps in knowledge

**Fig. 1:**
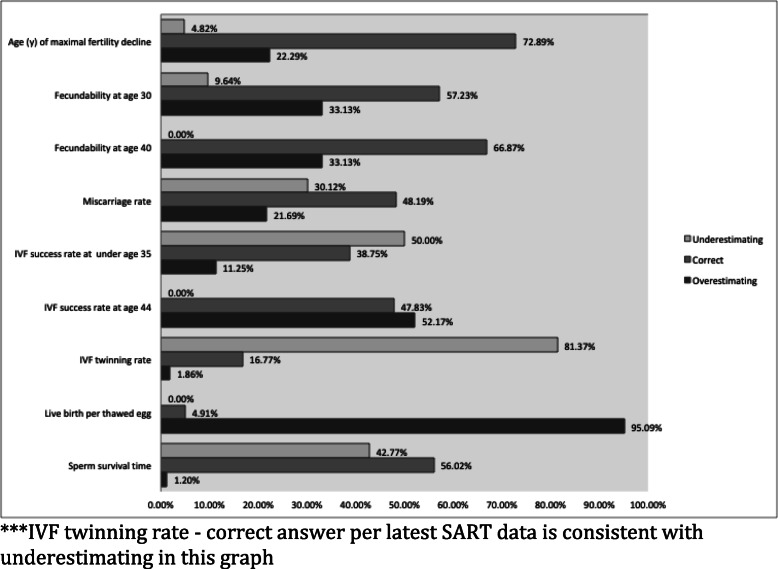
Fertility Items

Significant gaps in knowledge were noted (Table 2 and Fig. 1). Many answered incorrectly regarding fecundity, age of precipitous fertility decline, and In Vitro Fertilization (IVF) success rates. Fifty-nine percent incorrectly identified pregnancy rate per cycle for a woman under 35 years old under going in vitro fertilization. Forty-three percent did not know the average survival time of normal sperm in the female reproductive tract. Only 73% knew that the male partner’s age affected fertility, 48% knew that moderate alcohol consumption did not affect fertility, and 70% knew that using certain types of sexual lubricants affects fertility.

**Table 2:** Risk Factors

Risk factor	Correct Answer	N (%) answering correctly
Smoking	TRUE	163 (98%)
Being underweight	TRUE	165 (99%)
Prior use of oral contraceptive pills	FALSE	163 (98%)
*Gonorrhea* or *Chlamydia* infection	TRUE	161 (97%)
Occasional caffeine intake	FALSE	154 (93%)
Obesity	TRUE	165 (99%)
Safely-conducted pregnancy termination	FALSE	162 (98%)
Using certain types of sexual lubricants	TRUE	116 (70%)
Moderate alcohol consumption	FALSE	79 (48%)
Male partner’s age	TRUE	122 (73%)

## Discussion

Substantial gaps exist in fertility knowledge among OB-GYN residents. Without a strong understanding of this topic, they may not be prepared to properly counsel patients and have family planning discussions during routine visits, an essential part of well woman care. Our findings should be viewed by residency program directors as a starting place to encourage more exploration of this gap in knowledge in their own programs.

Knowledge of fertility does not change throughout residency training, with this study demonstrating consistent gaps in fertility knowledge. Knowledge during residency is only slightly higher than mean scores found in prior research in Internal Medicine residents (65%), as well as a cohort of female medical students and obstetrics and gynecology residents and fellows (64.9%) [14, 15]. In prior studies, the median score for reproductive-aged women was 16/29 (55.2%) and in medical trainees the median score was 19/29 (65.5%) [14]. Lack of time dedicated to education on this topic during both medical school and residency may be contributing to the patterns seen in physicians’ childbearing choices. This may also cause insufficient counseling and engagement of patients on family planning choices. Less than a quarter of reproductive-aged women have had discussions regarding reproductive health with their health care providers [2]. Although it is encouraging that the majority of residents stated that they discuss fertility with their patients, and the majority feel comfortable having this discussion, it remains concerning that several key areas of misinformation were identified, particularly regarding the overestimation of ART success rates.

One area that the survey may not be reflective of current practice is twinning rate. Correct answer in the FIT-KS survey was coded as 21–35% twinning rate, but most recent SART data is closer to 12%, making the majority of resident respondents correct according to the most recent data.

As women choose to delay childbearing, they will increasingly rely on ART, and should be sufficiently counseled on success rates that also decrease with aging [4]. In this study, there was a large overestimation of success of IVF after the age of 44. The misconception that ART can be used successfully with a couple’s own genetic material to compensate for the natural decline of fertility with aging should be counteracted by consistent discussion well before women reach the natural limits of their reproductive capacities [16]. In order for gynecologists to lead these conversations with their future patients, they must receive adequate training on fertility counseling during training.

This study has a number of strengths, including using a newly validated survey, the FIT-KS, which was developed for use in physician populations. We also avoided negative reporting bias by including several analysis that were performed which upheld the null hypothesis, that is differences between the groups did not have an effect on their FIT-KS scores. To avoid selection bias, we sent the FIT-KS survey instrument to all OB-GYN residency directors in the country (*N* = 255).

Limitations of this study include the response rate. Our response rate calculation is imprecise as the exact number of residents who actually received the recruitment email from their residency directors is unknown. If all residents did have access to the survey, the response rate would be approximately 4%, a magnitude consistent with other published survey research of obstetrics and gynecology residents through email recruitment - at 2.2 and 5% respectively [17, 18]. It is impossible to ensure that the survey was received by all of the intended recipients. Although we attempted to avoid selection bias by recruiting through email, some bias may be in this data set by the self-selection of participants as those who are willing to spend the time to answer a survey may have different practices then those who do not, which was not measured. It is an interesting thought experiment to consider whether these responses were representative of the population sampled - certainly those who were more likely to answer could possibly be assumed to also have more interest in and motivation for learning about reproductive endocrinology and infertility. This would make the data overestimate the true knowledge in this population, as residents with an interest in reproductive endocrinology and infertility would likely have a higher knowledge base then those who have other interests. This, therefore would make the situation even more dire than it even appears in this sample. We also do not know if a single or several residency programs were overrepresented in the sample as we do not know which programs had multiple residents respond.

There was also a small over-representation of women in the sample, as approximately 85% of residents in Obstetrics and Gynecology are female compared to 91% in our sample [19]. Selection bias or women’s concerns about their own fertility may be a cause of this over-representation. This warrants further exploration in future studies. Although not all obstetrics and gynecology residents will desire to have children, those who do may not be adequately prepared to make informed decisions about their future childbearing plans. Of course there are many other factors that encourage physicians to delay pregnancy including but not limited to career plans, availability of childcare, financial burden of children during residency. If this lack of fertility knowledge encourages physicians to delay pregnancy (for instance beyond the completion of training) they may be inadvertently reducing their chances at childbearing due to natural decline in fertility.

Going forward, additional research should be performed both on obstetrics and gynecology residents and other medical specialties to further elucidate knowledge of age-related fertility decline, as our limited residency response rate limits the generalizability of this data. Additionally, the answers were not updated to reflect the latest SART data regarding infertility technology rates of success, which should be done for future uses of this survey. Data could also be collected regarding training program location, IVF/fertility program in house, fellowship program attached to residency, when Reproductive Endocrinology and Infertility (REI) rotations occur, and total time on REI rotation. Additional information should be gathered about the different REI curriculums at residency programs as they vary greatly throughout the country.

Moreover, interventions, such as an online didactics curriculum on natural fertility and age related fertility decline should be developed for use in residency programs and as continuing medical education to increase knowledge in this area. Data should be collected from these endeavors such as CREOG scores prior to intervention, a pre-test, a post-test several months after intervention to gauge retention of the subject matter, and the next year’s CREOG score.

## Conclusion

Knowledge of age related fertility decline among obstetrics and gynecology residents is limited. Misconceptions about natural fertility, risk factors, and success of treatments may significantly affect the lives of both physicians and the patients they treat. The FIT-KS instrument is a valuable tool that should be continued to be used in the future in both physician and general populations, however going forward, alternative recruitment methods will assist in generating more useful data for analysis.

## Supplementary Information

**Additional file 1.** SURVEY + FIT-KS Instrument.

## Data Availability

The data that support the findings of this study are available from the corresponding author, LMR upon reasonable request. Presented as a oral presentation at the American Society for Reproductive Medicine Scientific Congress and Expo on October 16, 2019.

## Abbreviations

ANOVA: Analysis of Variance
ART: Assisted Reproductive Technologies
FIT-KS: The Fertility and Infertility Treatment Knowledge Score
IVF: In Vitro fertilization
OB-GYN: Obstetrics and Gynecology
PGY: Post Graduate Year
REI: Reproductive Endocrinology and Infertility
SART: Society for Assisted Reproductive Technology
