# Supplementary material for: A cross-sectional survey of fertility knowledge in obstetrics and gynecology residents
Source: Fertil Res Pract. 2020 Dec 9;6:22. doi: 10.1186/s40738-020-00091-2 (PMC7724860; doi:10.1186/s40738-020-00091-2)
Supplement: Supplementary file 1 — Additional file 1. SURVEY + FIT-KS Instrument. [file 40738_2020_91_MOESM1_ESM.docx]

**SURVEY + FIT-KS Instrument**

Fertility Knowledge Survey

1) What is your sex?

Male

Female

2) What is your age?

18-25

26-30

31-35

36-40

41-45

3) What is your current level of training?

PGY1

PGY2

PGY3

PGY4

PGY5

PGY6

PGY7

Prefer not to say

4) What is your specialty?

Anesthesiology

Dermatology

Emergency Medicine

Family Medicine

Internal Medicine

Neurology

Neurosurgery

Obstetrics and Gynecology

Ophthalmology

OMFS

Orthopedic surgery

Otorhinolargology

Pathology

Pediatrics

PMR

Plastic surgery

Psychiatry

Radiology

Surgery

Urology

Vascular Surgery

**The following questions relate to natural fertility:**

5) At what age are women most fertile?

□ 12-19

□ 20-29*

□ 30-39

□ 40-49

6) Over which age range does a woman’s ability to get pregnant decline most precipitously?

□ 25-29

□ 30-34

□ 35-39*

□ 40-45

7) Over the course of 1 month, what is the percent chance that a 30yo woman who is trying to get pregnant will get pregnant?

□ 10%

□ 20%*

□ 30%

□ 40%

8) Over the course of 1 month, what is the percent chance that a 40yo woman who is trying to get pregnant will get pregnant?

□ ≤5%*

□ 6-10%

□ 11-15%

□ 16-20%

9) On average, for a woman in her peak reproductive years, what is the percent chance that a pregnancy (recognized or unrecognized) will end in a miscarriage?

□ ≤5%

□ 6-15%

□ 16-25%*

□ 26-35%

10) A woman and a man can both contribute to a couple's infertility:

□ True*

□ False

11) A man’s age is a factor that affects a couple’s fertility:

□ True*

□ False

12) Having less than 9 periods in a year can be normal for some women and doesn’t require any further evaluation:

□ True

□ False*

13)What is the average survival time of normal sperm in the female reproductive tract?

□ 12-24 hours

□ 24-48 hours

□ 3-5 days*

□ 6-9 days

14) When is the optimal time to have sexual intercourse in order to get pregnant?

□ Right before the period starts

□ First few days of the period

□ About halfway through the cycle*

□ It doesn’t matter

15) Where does fertilization most commonly occur?

□ In the uterus

□ Inside the ovaries

□ On the surface of the ovaries

□ In the Fallopian tubes*

16) How many eggs are typically released per cycle?

□ 1*

□ 2

□ 3

□ 4

**The following are likely to decrease a woman’s chance of fertility:**

True False

17) Smoking……………………………………………. □* □

18) Occasional caffeine intake………………………. □ □*

19) Moderate alcohol consumption………………….. □ □*

20) Safely-conducted pregnancy termination …….... □ □*

21) Obesity……………………………………………... □* □

22) Gonorrhea or Chlamydia infection………………. □* □

23) Prior use of oral contraceptive pills……………… □ □*

24) Being underweight due to frequent exercise

or limited caloric intake…………………………… □* □

25) Using certain types of sexual lubricants…………. □* □

**The remainder of questions relate to infertility treatment:**

26) In vitro fertilization (IVF) refers to an infertility treatment in which:

□ A thin catheter is used to deposit a man’s sperm past the cervix directly into the uterus

□ A man’s sperm and a woman’s egg are combined inside a laboratory and the resulting embryo is transferred into the uterus*

□ When a woman carries a pregnancy for another couple who cannot get pregnant

Surgery is performed to harvest sperm from the man

**The following 3 questions refer to the most recent national statistics published by the Centers for Disease Control & Prevention and the Society for Assisted Reproductive Technology:**

27) For a woman under 35 years old, undergoing IVF with her own eggs, what is the pregnancy rate per cycle?

□ ≤5%

□ 6-20%

□ 21-40%

□ 41-60%*

□ ≥ 60%

28) For a woman over 44 years old, undergoing IVF with her own eggs, what is the pregnancy rate per cycle?

□ ≤5%*

□ 6-20%

□ 21-40%

□ 41-60%

□ ≥ 60%

29). In women who are undergoing IVF, what is the percent of pregnancies that result in twins?

□ ≤5%

□ 6-20%

□ 21-35%*

□ 36-45%

30) What is the average cost of an IVF cycle in the USA?

□ $5,000

□ $12,000*

□ $20,000

□ $50,000

31) Intrauterine insemination (IUI) refers to a treatment in which:

□ A thin catheter is used to deposit a man’s sperm past the cervix directly into the uterus*

□ A man’s sperm and a woman’s egg are combined inside a laboratory and the resulting embryo is transferred into the uterus

□ Sperm are deposited directly into the vagina (“turkey baster”)

□ Surgery is performed to harvest sperm from the man

32) Egg cryopreservation (freezing) refers to an infertility treatment in which:

□ A single sperm is injected into an egg to preserve the egg’s integrity

□ A man’s sperm and woman’s egg are combined inside a laboratory and then frozen

□ Strips of ovarian tissue are surgically removed and frozen

□ Eggs are frozen following ovarian stimulation and egg retrieval*

33) As per the largest published studies, when using frozen eggs from women less than 37 years old, what is the live birth rate per thawed egg?

□ ≤10%*

□ 11-15%

□ 16-20%

□ 21-25%

34) Are you concerned about your own future fertility?

Yes

No

35) Do you ever discuss fertility with your patients?

Yes

No

36) Do you feel comfortable answering patient’s questions about fertility?

Yes

No

37) Do you currently have any children?

Yes

No

38) What is your desired number of children?

Blank

39) What is your ideal age to have children?

Blank

40) What would you do if you and your partner were not able to get pregnant?

Undergo fertility medications

Undergo IVF

Adoption

Choose not to have children
